# Point-of-care Ultrasound Aiding in the Diagnosis of Herlyn-Werner-Wunderlich Syndrome

**DOI:** 10.5811/cpcem.2017.7.34089

**Published:** 2017-10-18

**Authors:** Robert Ellspermann, Caroline Sirhari, Ethan Chapin, Mathew Nelson

**Affiliations:** * North Shore University Hospital, Department of Emergency Medicine, Manhasset, New York; † University of Florida, Department of Emergency Medicine, Gainesville, Florida; ‡ Jupiter Medical Center, Department of Emergency Medicine, Jupiter, Florida; § North Shore University Hospital, Department of Emergency Medicine, Division of Emergency Ultrasound, Manhasset, New York

## Abstract

We present a case of a 12-year-old female with a history of congenital solitary kidney presenting to an academic pediatric emergency department (ED) in acute abdominal pain. Using ultrasound as the initial diagnostic modality, the patient was found to have Herlyn-Werner-Wunderlich syndrome (HWWS), an abnormal development of the Müllerian system during embryogenesis resulting in obstructed hemivagina with resulting hematometrocolpos. The patient presented with undifferentiated abdominopelvic pain, and in the course of the ED workup was diagnosed with a disorder infrequently encountered by emergency physicians. We present a case of markedly abnormal point-of-care ultrasound findings prompting additional studies, ultimately leading to a diagnosis of HWWS during the initial ED visit.

## INTRODUCTION

An exceedingly rare disorder, Herlyn-Werner-Wunderlich syndrome (HWWS) is characterized by a triad of uterus didelphys, obstructed hemivagina, and unilateral renal agenesis. The pathogenesis of the disease arises from an abnormal development of the Müllerian system during embryogenesis resulting in an obstructed hemivagina with resulting hematometrocolpos. First described in 1922, it has an incidence of 1:20,000.^1^,^2^ The mean age of onset in patients with complete obstruction of the hemivagina is 13 years, with average time of four months from menarche to symptoms.^1^ However, cases in which the hemivagina is incompletely obstructed can have a much later onset of symptoms and diagnosis.^1^ Due to its rarity, there is a lack of awareness of HWWS, particularly among emergency physicians (EP). We present a case in which early diagnosis, made possible with point-of-care ultrasound (POCUS), led to prompt treatment and avoidance of further complications of this uncommon disease.

## CASE REPORT

A 12-year-old female presented to an academic emergency department (ED) with abdominal pain that was reported to have started one hour prior to arrival. The pain was localized to the lower abdomen, rated at 4/10 – 6/10, was constant, aching, persistent, and non-radiating. The patient denied fever, nausea, vomiting, diarrhea, vaginal bleeding or discharge. She had not taken anything to relieve the pain and it was not better with time. Her last bowel movement was the day before and typical. Further review of systems failed to elicit any dysuria, urinary frequency or urgency. Last menstrual period began one week previously and finished three days prior to presentation. Menarche was reported to be age 10 and menstrual cycles had been consistent monthly and occurring regularly around a 28-day cycle. When questioned, the patient recalled similar pain but to a lesser extent at four days after completion of menstruation the previous month. When questioned privately, the patient denied any sexual activity.

Vital signs on arrival were blood pressure 111/68 mmHg, heart rate 78 beats per minute, respiratory rate 22 breaths per minute, pulse oxygenation 95% on room air, and temperature 98.2 °F orally.

On exam, the patient appeared comfortable and well developed, in no apparent distress, and was alert and oriented to her surroundings. Her abdominal exam was significant for mild middle and right lower quadrant tenderness (slightly lateral from midline) that was not reproduced on subsequent exams. Her abdominal exam was negative for Rovsing’s sign and McBurney’s point tenderness. The patient exhibited no rebound or guarding, no costovertebral tenderness, and there were no other significant findings. The patient’s external genitalia were at normal development for a 12-year-old female, and no abnormalities were noted.

At this point the differential diagnosis consisted of ovarian cyst, ruptured hemorrhagic ovarian cyst, ovarian torsion due to the severity of pain, urinary tract infection and, less likely, appendicitis due to the absence of fever, chills, nausea, and vomiting. The history and exam along with our differential gave concern for the need of surgical therapy. In attempts to reduce radiation exposure and expedite disposition and treatment, a point-of-care ultrasound (POCUS) was ordered along with complete blood count, complete metabolic panel, prothrombin time, international normalized ratio (INR), partial thromboplastin time, urinalysis, and a type and screen. POCUS is readily available and frequently used in our ED and was available by an EP at the time of this patient’s presentation. It was performed to evaluate for ovarian torsion, an ovarian cyst, a ruptured ovarian cyst, or less likely appendicitis.

A trans-abdominal POCUS showed a hypoechoic structure of indeterminate etiology in the right lower quadrant, to the right of the uterus, filled with hypoechoic material (Image, Video). There was consideration for fluid collection, hemorrhage, and even for malformed kidney in the pelvis. These findings prompted a comprehensive radiologic US, which led to the diagnosis.

Laboratory findings were as follows: white blood cells 10.6 K/uL, hemoglobin/hematocrit 12.5 g/dL/36.7 %, platelets 275 K/uL, INR 1.03 ratio, sodium 144 mmol/L, potassium 4.3 mmol/L, chloride 107 mmol/L, carbon dioxide/bicarbonate 24 mmol/L, blood urea nitrogen 14 mg/dL, creatine 0.56 mg/dL, glucose 118 mg/dL. The urinalysis was negative (specifically for nitrite and leukocyte esterase concentration), as was the urine pregnancy test.

Obstetrics and gynecology was consulted to evaluate the patient in the ED after the radiology US was performed. With a probable diagnosis in hand, the patient was discharged home to follow up with a pediatric gynecologist for operative evacuation of the hematocolpos and repair of her blind-ending hemivagina.

## DISCUSSION

This is a near-classic presentation of HWWS in a post-menarche female in early adolescence presenting with symptoms of abdominal pain. Patients are usually diagnosed after menarche, although some cases of diagnosis in early childhood and even in utero have been reported.^3^,^4^ Some cases have also been reported in which diagnosis occurs during patients’ pregnancies or secondary to infertility struggles in adulthood.^5^,^6^ As in this case, symptoms are often related to obstruction of the hemivagina, as endometrial effluent accumulates in a blind-ended pouch. However, despite her completely obstructed hemivagina, this patient did not present until two years after menarche, a relatively long time.

**CPC-EM Capsule**

What we already know about this clinical entity?

Herlyn-Werner-Wunderlich Syndrome, is a congenital disorder resulting in obstructed hemivagina with resulting hematometrocolpos. It is characterized by a triad of uterus didelphys, obstructed hemivagina, and unilateral renal agenesis.

What makes this presentation of disease reportable?

In this case presentation, despite her completely obstructed hemivagina, this patient did not present until two years after menarche, a relatively long time – making the diagnosis much more difficult.

What is the major learning point?

With improved availability of point-of-care ultrasound, Emergency physicians are often scanning more frequently encountered, life threatening abnormalities. The use of ultrasound as a routine part of the abdominal exam increases the odds of identifying sonographically identifiable deviation from normal anatomy.

How might this improve emergency medicine practice?

Without the use of early ultrasound in patients’ presentation to the emergency department, there is risk for diagnostic delay, overuse of imaging requiring radiation or misdiagnosis.

Due to its rarity, HWWS is often diagnosed after considerable delay, frequently after previous misdiagnoses and invasive surgical procedures.^2^,^7^,^8^ The patient in this case had a known ipsilateral renal agenesis identified in childhood, but no further investigation was prompted at the time. The relative rarity of HWWS likely contributed to this, but in the presence of other urogenital anomalies, consideration for Müllerian abnormalities should be entertained.^9^ This will generally be done by an obstetrician/gynecologist, but in adolescent females in this age group who are not sexually active, it will often fall to the pediatrician to screen and to the EPs to diagnose once the patients become symptomatic.

Because the embryologic origins of the female reproductive system and the urinary system develop in tandem, the anomalies in HWWS are ipsilateral. The paramesonephric (Müllerian) and mesonephric ducts give rise to the superior two-thirds of the female reproductive tract and the urinary system, respectively. Therefore, the blind hemivagina and renal agenesis occur on the same side, as the fusion that normally joins one side of these bilateral systems to their contralateral counterparts fails to occur. Right-sided abnormalities are more common than left-sided abnormalities; we do not know the cause for this predominance.^10^ Right-sided abnormalities account for 60–70% of HWWS, thus expanding the already extensive differential for right-sided abdominal pain in a young female patient in the ED.^1^,^10^,^11^

In an emergency setting, rare diseases like HWWS are usually absent from the common differential of undifferentiated abdominal pain in adolescent females. Abdominal pain in these patients is often investigated with serial abdominal exams, formal radiologic US, or computed tomography where clinically warranted. The differential generally includes appendicitis, ovarian torsion, ruptured ovarian cyst, and pelvic inflammatory disease among others. However, improved availability of POCUS in emergency medicine is a key tool for improved identification of other, less common diagnoses, including this rare abnormality. Although EPs are often scanning more frequently encountered, life- threatening abnormalities, use of US as a routine part of the abdominal exam increases the odds of identifying this sonographically identifiable deviation from normal anatomy.

In this case, POCUS did not make the definitive diagnosis; rather, marked anomalies easily detected on POCUS prompted further imaging studies. A radiologic US ordered shortly after POCUS provided the definitive diagnosis. Without the use of US early in presentation to the ED, there is risk of diagnostic delay (potential for prolonged for worry, pain, and suffering in a child and her family), iatrogenic harm (in the form of radiation), or misdiagnosis. While magnetic resonance imaging is considered the gold standard for imaging of Müllerian ductal anomalies, the availability and accuracy of US make it not only ideal for initial screening and assessment, but also for definitive diagnosis in cases such as this presentation.^12^,^13^

## CONCLUSION

This diagnosis previously was managed with radical surgical management, often hysterectomy or partial hysterectomy.^14^ However, with current management and successful surgical resection of the uterine septum and excision of the blind hemivagina, HWWS has an excellent prognosis for preserved fertility.^1^ Failure to expeditiously and accurately diagnose this condition can lead to both short-term and long-term complications, including infection, pyocolpos, urinary retention, pelvic mass effect, and infertility.^5^,^15^,^16^ The use of POCUS as a diagnostic tool in this case led to prompt diagnosis and resection of the septum within one week.

## Supplementary Information

Video Trans-abdominal ultrasound demonstrating findings consistent with Herlyn-Werner-Wunderlich syndrome. While scanning through the region of the right adnexa anterior to the ovary you see a blind hemivagina in display, indicated by the thin arrow, and the uterus with the thick arrow. Note that both the hemivagina and uterus are filled with hypoechoic material, the hemivagina separated with a hyperechoic wall definitively separating it from the uterus. The thin arrow is pointing to the hematocolpos, which was noted to displace the normal anatomy on ultrasound.

## Figures and Tables

**Image:**
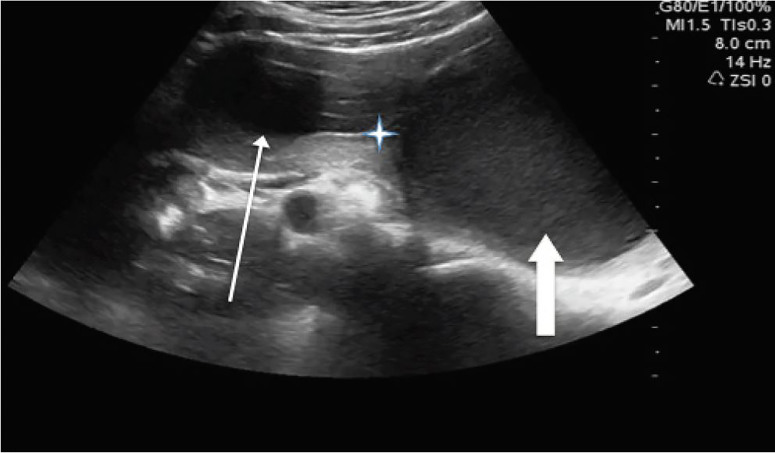
Phased-array, transverse ultrasound scan in the right adnexal region demonstrating a blind hemivagina (thin arrow) and the uterus (thick arrow). Both structures are filled with hypoechoic material and separated by a hyperechoic wall (star).
